# Combined effect of beet powder and lentil flour as a partial nitrite substitute on physicochemical, texture and sensory characteristics, color, and oxidative stability of pork bologna

**DOI:** 10.1111/1750-3841.16302

**Published:** 2022-09-19

**Authors:** Jihan Kim, Phyllis J. Shand

**Affiliations:** ^1^ Smart Foods Innovation Centre of Excellence AgResearch Ltd. Palmerston North New Zealand; ^2^ College of Agriculture and Bioresources University of Saskatchewan Saskatoon Saskatchewan Canada

**Keywords:** beet powder, color, consumer sensory evaluation, lentil flour, nitrite, pork bologna

## Abstract

The combined effect of beet powder (BP; 0.1%, 0.3%, and 0.5%) and 6% lentil flour (LF) as a partial nitrite substitute on quality attributes of pork bologna incorporated with 7.5% mechanically separated pork during 12 weeks of cold storage was evaluated. A randomized block experimental design with nine treatments and five storage times (0, 3, 6, 9, and 12 weeks) was used for pH, thiobarbituric acid reactive substance, protein oxidation (carbonyl and sulfhydryl), and color parameters. Bologna preparation at a pilot plant scale was replicated three times. LF addition resulted (*p* < 0.05) in higher viscosity, emulsion stability, and lower expressible drip. Together BP and LF decreased protein oxidation during storage but were not as effective as nitrite with respect to lipid oxidation. Bologna with BP addition showed lower lightness and higher redness, whereas LF increased lightness and decreased redness. A consumer panel rated color acceptability of bologna lower with LF addition, whereas BP raised color acceptability. However, addition of the highest BP level alone to the bologna was negatively perceived as a result of the low acceptability of purge color of the vacuum‐packaged bologna slices. Consequently, the combination of BP and LF could be used to improve eating quality and stability attributes when used as a potential nitrite substitute.

## 1 INTRODUCTION

Sodium nitrite is prevalent as a functional ingredient for meat products as a colorant, an antioxidant, and due to its inhibition of microbial growth (Majou & Christieans, 2018). However, there are some concerns that residual nitrite may generate carcinogenic compounds under gastrointestinal conditions, but it still remains widely used in cured meats (Honikel, 2008; Santarelli et al., 2010). Thus, to replace the use of nitrite in meat products, studies have explored various replacement ingredients of natural origin (de Almeida et al., 2015; Djeri & Williams, 2014).

The potential functional effect of beet powder (BF) as a replacer for artificial ingredients such as colorants and antioxidants for meat products has been researched under different approaches. Beetroot powder contains two representative betacyanins (red) and betaxanthins (yellow) pigments that are classified as betalain pigments (Strack et al., 2003). Ravichandran et al. (2013) proposed the use of betalain as an effective strategy to preserve foods. In addition, beetroot powder protected fresh pork sausage quality from deterioration and preserved sensory acceptability under lighting display (Martínez et al., 2006). Color acceptability of cooked sausage formulated with 0.5% and 1.0% levels of BF was enhanced, whereas the BF addition was not helpful to protect the products against oxidative damage (Jin et al., 2014). Based on the previous studies, antioxidant properties of BF can be enhanced by combination with natural plant sources (Ozaki et al., 2021; Pennisi et al., 2020).

Natural alternative sources of nonmeat ingredients such as soy‐based products have been used in processed meat products as binders and extenders to improve textural properties and cooking yield due to their high protein and starch contents (Serdaroğlu et al., 2005; Shariati‐Ievari et al., 2016). Pulses such as lentil and pea are currently gaining popularity as binders (Chigwedere et al., 2022). One of the major concerns for using some pulse‐based binders was higher susceptibility to oxidation because of endogenous lipoxygenase, but infrared heat treatment has been applied to pulse binders for inactivation of this enzyme as well as enhancement of consumer acceptability when heat‐treated chickpea or lentil flours (LFs) were added to meat products (Shariati‐Ievari et al., 2016; Sharima‐Abdullah et al., 2018). Further, infrared‐heated LF increased lipid stability in chicken sausages, primarily made from mechanically separated chicken as a source of easily oxidizable meat (Pathiraja & Shand, 2018).

The use of LF together with BF for pork bologna incorporated with mechanically separated pork may enhance oxidative stability and improve sensory characteristics in the absence of sodium nitrite. The novelty of the work presented here lies in it being the first application of the combination of BF with LF as a sodium nitrite substitute in meat products. In addition, we wanted to investigate consumer perception of the color of the cooked bologna and of the color pigment released in purge of vacuum‐packaged meat product. Therefore, this research examined the effect of the combination of different levels (0.1%, 0.3%, and 0.5%) of BF and LF (0 and 6%) substituting for sodium nitrite on physicochemical, textural, and sensory characteristics, oxidative stability, and eating quality of vacuum‐packaged pork bologna incorporated with mechanically separated pork.

## 2 MATERIALS AND METHODS

### 2.1 Raw materials and manufacturing

Vacuum‐packaged pork shoulder picnic muscles, mechanically separated pork, and back fat were collected from Maple Leaf Foods Inc. (Manitoba, Canada) on three occasions. The connective tissue and excess fat of muscles were trimmed and then the meat was ground using a grinder with 8‐mm plate. The ground meat for each of the three replications was immediately stored at −30°C before the processing. The control formulation consisted of 45.0% pork picnic shoulder, 7.5% mechanically separated pork, and 12.5% back fat. Nonmeat ingredients were also used to formulate bologna, including ice water, sodium chloride, sodium triphosphate, Prague powder containing sodium nitrite (to deliver 156 ppm), sodium erythorbate, and German‐style wiener seasoning. Part of the water content was replaced by the different levels of LF (0% and 6%) and BF (0.1%, 0.3%, and 0.5%) according to the product formulation (Table 1). The 6% lentil addition was determined based on a preliminary study that evaluated functionality of lentil in mechanically separated meat systems, as a reported by Pathiraja and Shand (2018). Another preliminary test was also conducted to investigate adequate BF levels for nitrite‐derived color replacement in laboratory‐scale meat emulsions fortified with lentil. The heat‐treated LF (NutraReady™, Saskatoon, SK, Canada) was commercially obtained from InfraReady Products Ltd. (Saskatoon, SK, Canada), and the BF was obtained from Malabar Ingredients Inc. (Mississuaga, ON, Canada).

**TABLE 1:** Formulations of the experimental pork bologna

	Non‐lentil flour addition				Lentil flour addition				
	CON	0.1B	0.3B	0.5B	L0.0B	L0.1B	L0.3B	L0.5B	LCON
Pork	45.00	45.00	45.00	45.00	45.00	45.00	45.00	45.00	45.00
MSM	7.50	7.50	7.50	7.50	7.50	7.50	7.50	7.50	7.50
Back fat	12.50	12.50	12.50	12.50	12.50	12.50	12.50	12.50	12.50
Ice water	32.25	32.82	32.02	31.82	26.32	26.22	26.02	25.82	26.25
Sodium chloride	1.56	1.78	1.78	1.78	1.78	1.78	1.78	1.78	1.56
Sodium triphosphate	0.30	0.30	0.30	0.30	0.30	0.30	0.30	0.30	0.30
Prague powder^c^	0.24	0.00	0.00	0.00	0.00	0.00	0.00	0.00	0.24
Seasoning	0.60	0.60	0.60	0.60	0.60	0.60	0.60	0.60	0.60
Beet powder	0.00	0.10	0.30	0.50	0.00	0.10	0.30	0.50	0.00
Lentil flour	0.00	0.00	0.00	0.00	6.00	6.00	6.00	6.00	6.00
Sodium erythorbate	0.05	0.00	0.00	0.00	0.00	0.00	0.00	0.00	0.05
Total	100	100	100	100	100	100	100	100	100

^a^ Pork, pork shoulder (59.03% moisture, 15.75% fat, and 24.22% protein).

^b^ MSM, mechanically separated pork (60.66% moisture, 23.76% fat, and 14.58% protein).

^c^ Prague powder containing 6.4% sodium nitrite.

^d^ Seasoning, German‐Style Wiener seasoning (mustard, paprika, dextrose, and spice extractions).

Processing was conducted with pilot‐scale equipment in the meat pilot plant at the University of Saskatchewan. For each of three replicates (prepared on different days), ground pork, mechanically separated pork, and back fat were homogenized with the nonmeat ingredients using a bowl chopper (RMF, Grandview, MO, USA) for 4 min. The homogenates were emulsified twice using an emulsion mill (Type 1E‐75F, Alexanderwerk, Remschied, Germany). The air in the emulsion was removed by pulling a vacuum (2×) on the meat batter in a single‐chamber vacuum packaging machine (Model 550A, Sipromac, Quebec, Canada) and then the batter was stuffed into waterproof casing (63 mm diameter) with a Handtmann VF80 stuffer (Biberach/Riss, Germany). The cooking procedure was conducted as described by Shand (2000). Briefly, the stuffed casings were placed in a 1°C cooler overnight. Then the chubs were cooked in a water bath using a staged process to reach a core temperature of 72°C. The cooked samples were cooled in cold water and placed at 4°C overnight. Two chubs per treatment were opened for cook loss determination. Then, samples were sliced using a deli meat slicer for physical and textural properties, and vacuum packaged (Cryovac barrier bags, oxygen transmission rate 3–6 cc O_2_/m^2^/24 h) for pH, color, and oxidative stabilities measurements in the dark during 12 weeks of storage time. Samples were collected at 3‐week intervals for 12 weeks with storage at 4°C (0, 3, 6, 9, and 12 weeks).

### 2.2 Proximate composition, pH, and instrumental color measurement

Proximate composition (moisture, crude fat, crude protein, and ash) of samples was determined by standard methods of AOAC (1990). For pH measurement, 20 g of sample was homogenized with 80 ml distilled water by using a stomacher (Stomacher^®^ 400 Lab Blender Series, Seward, AK, USA). The pH was then measured with a pH meter (Accumet AR 15, Fisher Scientific Inc., Pittsburgh, PA, USA). According to the Commission Internationale de l'Elcaorage (CIE) color system, the color of two uncooked and cooked samples at 0 days (CIE L*, lightness; CIE a*, redness; and CIE b*, yellowness) was measured in duplicate using a Hunterlab Miniscan XE (Hunter, Reston, VA, USA) with illuminant A and observer 10. The color of two packages per treatment stored in the dark at 4°C was measured every 3 weeks in duplicate. The instrument was standardized using white and black tiles. In addition, a pink color tile was periodically measured to check for instrument stability before measuring samples. In these assays, two samples in each of the three replications of the experiment were analyzed in duplicate and the average of each treatment was used in the statistical analysis.

### 2.3 Cooking loss, purge loss, and expressible drip

To determine cooking loss, two raw chubs per each treatment were weighed. After cooking, waterproof casings were removed and then the cooked chubs were weighed. The cooking loss was calculated as a percentage of raw chub weight before cooking. The measurement of purge loss was described by Shand (2000). Six slices with 3 mm thickness of samples were weighed and then vacuum packed. The packages were placed in dark condition for 14 days at 4°C. After 14 days of storage time, the slices were blotted and weighted. Expressible drip was measured in duplicate as described by Shand (2000). A sample of bologna (1.5 ± 0.3 g) was placed in the thimble and was centrifuged for 15 min at 2400 × *g* (centrifuge 5810 R, Eppendorf, Germany). The expressible drip was expressed as percentage of weight loss of samples after centrifugation.

### 2.4 Emulsion stability

Emulsion stability of samples was determined in duplicate as described by Colmenero et al. (2005). Ten grams of meat batter was centrifuged at 2500 × *g* for 5 min in quadruplicate. The samples were then placed in a water bath at 80°C for 60 min. The sample tubes were placed upside down for 45 min to release the exudates. The total amount of released fluids was calculated as a percentage of the sample weight. To determine the released fat and moisture loss, the released fluids were placed in a drying oven at 105°C overnight. The calculations were expressed as below:

$$\begin{array}{ccc} & & \mathrm{Total}\;\mathrm{expressible}\;\mathrm{fluid}\;\mathrm{separation}\;\;\left(\%\right)\\ & & =\frac{\mathrm{Weight}\;\mathrm{of}\;\mathrm{total}\;\mathrm{fluid}\;\mathrm{released}\left(g\right)}{\mathrm{Sample}\;\mathrm{weight}\;\left(g\right)}\;\times 100, \end{array}$$

$$\mathrm{Fat}\;\mathrm{loss}\;\;\left(\%\right)=\frac{\mathrm{After}\;\mathrm{drying},\;\mathrm{the}\;\mathrm{weight}\;\mathrm{of}\;\mathrm{tube}\;\mathrm{with}\;\mathrm{fluid}\;\left(g\right)-\mathrm{the}\;\mathrm{weight}\;\mathrm{of}\;\mathrm{tube}\left(g\right)}{\mathrm{Sample}\;\mathrm{weight}\;\left(g\right)}\times 100,$$

$$\mathrm{Moisture}\;\;\mathrm{loss}\;\;\left(\%\right)=\mathrm{Total}\;\mathrm{expressible}\;\mathrm{fluid}\;\mathrm{separation}-\mathrm{Fat}\;\mathrm{loss}.$$

The analysis was performed on two samples in each of the three replications of the experiment.

### 2.5 Viscosity

Each meat batter was stuffed into a plastic cup and its viscosity was measured in duplicate after overnight storage at 4°C. The viscosity measurement was conducted by using a Brookfield Synchro‐Lectric viscometer (Model RVT, Brookfield Engineering Stoughton, MA, USA) set at 10 rpm and the data were expressed in centipoise. Two samples in each of the three replications of the experiment were analyzed in duplicate and the average of each treatment was used in the statistical analysis.

### 2.6 Textural measurements

Two cores (33 mm diameter and 30 mm height) per chub were collected using a coring device for textural profile analysis (TPA) and placed at room temperature for 30 min before analysis. Two chubs were used for each replicate. The condition for analysis was 15 mm of compression height (50% of sample height) for two cycles and 1.00 cm/s crosshead speed on a texture analyzer (TMS‐PRO texture press, Food Technology Crop., Rockville, MD, USA). TPA values (hardness: the peak force during the first compression cycle; cohesiveness: the ratio of the positive force area during the second compression portion to the positive force area during the first compression; springiness: the ratio between the time needed for the material to reach the maximum load since it starts to deform in the second compression and the time needed for the first compression; and chewiness: a parameter obtained multiplying the hardness times the cohesiveness time the springiness) were automatically calculated using the supplied software (Bourne & Comstock, 1981).

Eight core samples were obtained using a 12‐mm‐diameter coring device from a 30‐mm slab of two chubs per treatment. The ends of samples were glued to plastic disks with cyanoacrylate glue. Samples were trimmed into dumbbell shape with a bench‐top grinder and mounted on a torsion fixture on a Brookfield digital viscometer model (DV‐I, Gel Consultants Inc., Raleigh, NC, USA). Samples were twisted at 2.5 rpm until failure. Failure shear stress and strain were obtained using the provided software (Gelscan, Gel Consultants Inc.). Sixteen samples from two chub per treatment in each of the three replications of the experiment were analyzed and the average of each treatment was used in the statistical analysis.

### 2.7 Thiobarbituric acid reactive substances

The level of lipid oxidation of samples during 12 weeks of storage was measured by the modified thiobarbituric acid reactive substance (TBARS) procedure in accordance to Tarladgis et al. (1960). Three samples from each of two chubs collected at 3‐week intervals in each of the three replications of the experiment were analyzed and the average of each treatment was used in the statistical analysis. Five grams of sample was homogenized with 18 ml of distilled water. The homogenate was transferred into a Kjeldahl flask by washing with 37.5 ml of distilled water. Then, 1.5 ml of 4 N HCl solution was added into the flask with boiling stones and 1 ml of antifoam reagent. The final volume of the diluted homogenate was 50 ml. The samples were heated and then the first 50 ml of distillate was collected during the Kjeldahl distillation process. A 5‐ml aliquot of the distilled sample was mixed with 5 ml of 0.02 M 2‐thiobarbituric acid. Samples were placed in a boiling water bath for 35 min and then cooled in cold water for 10 min. The *K*‐value was calculated from the percentage of recovery and a standard slope (1,1,3,3‐tetraethoxypropane). The absorbance was recorded at 538 nm by using a UV spectrophotometer (UV 1800, Schimadzu, Japan). The TBA values are expressed as mg malondialdehyde (MDA) per kilogram sample.

### 2.8 Protein oxidation (carbonyl and sulfhydryl measurements)

Carbonyl and sulfhydryl contents were estimated from methods of Levine et al. (1994) and Jongberg et al. (2013) according to the procedures described by Vossen and De Smet (2015). The determination of carbonyl content was obtained according to the reaction between protein carbonyls and 2,4‐dinitrophenylhydrazine (DNPH). Sulfhydryl contents was determined using Ellman's test using 5,5′‐dithiobis (2‐nitrobenzoic acid). Both carbonyl and sulfhydryl contents were expressed as nmol per mg protein using a molecular extinction coefficient of 22,000 and 14,000/M/cm.

### 2.9 Sensory evaluation

For consumer sensory evaluation, a sixty‐member consumer panel was recruited from the College of Agriculture and Bioresources at the University of Saskatchewan. The consumer sensory panel protocol was approved by the University of Saskatchewan Behavioral Research Ethics Board (ID# 97). The sensory evaluation was conducted in two parts. Ten sessions were run with six consumers per session. In the first part, consumer panelists evaluated the nine packaged samples coded with three‐digit numbers in random order. Each treatment was prepared as for simulated retail display with a whole slice (2 mm thick) in vacuum pouches set on a white tray. Each panelist was asked to score the products for external, internal, and released purge color acceptability using a 6‐point hedonic scale where 1 = *dislike very much* to 6 = *like very much* in panel booths illuminated with white light (approximately 400 lux of warm white fluorescent light). Before asking the color acceptability, color descriptions of samples were also evaluated using a 6‐point scale where 1 = *gray brown*, 2 = *gray*, 3 = *dark pink*, 4 = *pale pink*, 5 = *bright pink*, and 6 = *bright red*.

In the second part, color, texture, juiciness, flavor, and overall acceptability of seven samples were evaluated using 6‐point hedonic scales, and of purchase intent using a 5‐point scale (1 = *definitely would not* to 5 = *definitely would*). To limit fatigue, the two treatments with 0.5% BF were not evaluated as these showed extremely low released purge color acceptability, with the released purge showing redness that would likely be perceived as that from under cooked meat. Panelists were presented with four cubes (1 × 1 × 1 cm) of bologna placed in covered cups coded with random three‐digit numbers and presented in random order. Unsalted crackers and water for palate cleansing were provided. A consumer survey about sodium nitrite consumption and awareness was also conducted. Consumer demand patterns for meat products were investigated such as price, color, meat species, absence of sodium nitrite, mechanically separated meat, and preservatives using 6‐point scales where 1 = *not at all* to 6 = *extremely important*.

### 2.10 Statistical analysis

A randomized block experimental design with nine treatments and five storage times (0, 3, 6, 9, and 12 weeks) was used for pH, TBARS, protein oxidation (carbonyl and sulfhydryl), and color parameters. Bologna preparation was replicated three times. Data were analyzed by using generalized linear mixed model to determine significant differences among treatments and storage effects as fixed effects, and the experiment replications as a random term (IBM SPSS 24.0). The effect of pork bologna formulation on other parameters was determined with one‐way analysis of variance (ANOVA) on means from each test with Tukey's test used to determine the statistical difference among the means (*P* < 0.05). Principal component analysis (PCA) was used to investigate the spatial configuration of technological and sensory attributes and the interrelationships between treatments. *Z*‐scores were used to standardize the dataset and minimize distortions caused by widely different attribute values.

## 3 RESULTS AND DISCUSSION

### 3.1 Physicochemical properties

Proximate composition, viscosity, cooking loss, expressible drip, and purge loss of pork bologna incorporated with LF and BP are reported in Table 2. The bologna products incorporated with 6% LF had lower moisture and higher protein contents than the other treatments (*p* < 0.001). Meat protein content should be similar among treatments as the same amount of meat was added. By calculation, the LF with a protein content of approximately 25% would have contributed 1.5% protein, which is in line with the proximate results. Similarly, the addition of grain binders increased protein and decreased moisture content of pork bologna (Shand, 2000). Incorporation of BP in the bologna products did not significantly affect proximate composition compared to the nitrite control. No significant effect on cooking loss due to LT and BP addition was detected. In the presence of LF, the bologna products showed higher viscosity of the raw batter and lower expressible drip of the cooked bologna than non‐lentil‐incorporated products (*p* < 0.001) and tended to have less purge (*p* = 0.067), showing good water holding capacity. Similar results have been reported for 2.5% and 5% chickpea flour addition to low‐fat pork bologna that reduced cooking loss and expressible moisture (Thushan Sanjeewa et al., 2010). The increased raw batter viscosity of the treatments with LF could be explained by the high protein and fiber contents (20.6% and 6.83%, respectively) according to de Almeida et al. (2015) and also the partial gelatinization of the starch in infrared‐heated LF (Dogan et al., 2013). However, we observed that cooking losses did not differ significantly among treatments as planned. In this study, bologna was cooked in moisture‐proof casings with sufficient meat protein to keep cook losses low.

**TABLE 2:** Proximate composition, raw batter viscosity, cooking loss, expressible drip, and purge loss of the pork bologna

Treatments	Proximate composition (%)				Viscosity (raw) (cps) 10^5^	Cooking loss (%)	Expressible drip (%)	Purge loss (%)
	Moisture	Fat	Protein	Ash				
CON	67.50 ± 0.41^b^	15.4 ± 0.239^c^	11.41 ± 0.09^b^	2.53 ± 0.03^bc^	0.85 ± 0.05^b^	1.14 ± 0.03^ns^	15.81 ± 0.25^a^	4.28 ± 0.75^ns^
0.1B	68.13 ± 0.17^b^	16.33 ± 0.28^abc^	11.92 ± 0.09^b^	2.46 ± 0.04^c^	0.85 ± 0.03^b^	1.14 ± 0.01	16.91 ± 0.41^a^	4.54 ± 0.83
0.3B	66.53 ± 1.04^b^	15.66 ± 0.24^bc^	11.46 ± 0.25^b^	2.47 ± 0.05^c^	0.85 ± 0.03^b^	1.09 ± 0.01	16.38 ± 0.15^a^	4.30 ± 0.65
0.5B	66.78 ± 0.30^b^	16.22 ± 0.90^abc^	12.10 ± 0.61^ab^	2.53 ± 0.01^c^	0.87 ± 0.03^b^	1.15 ± 0.02	16.51 ± 0.69^a^	4.22 ± 0.89
L0.0B	61.96 ± 0.24^a^	17.81 ± 0.47^abc^	13.22 ± 0.37^a^	2.64 ± 0.02^ab^	1.56 ± 0.05^a^	1.16 ± 0.05	9.98 ± 1.18^b^	2.57 ± 0.38
L0.1B	61.18 ± 0.54^a^	18.31 ± 0.33^a^	13.52 ± 0.15^a^	2.64 ± 0.03^a^	1.58 ± 0.02^a^	1.21 ± 0.07	10.87 ± 0.61^b^	2.49 ± 0.25
L0.3B	61.21 ± 0.09^a^	17.96 ± 0.53^ab^	13.04 ± 0.31^a^	2.67 ± 0.03^a^	1.62 ± 0.03^a^	1.19 ± 0.10	11.74 ± 0.45^b^	2.63 ± 0.29
L0.5B	62.49 ± 0.23^a^	17.84 ± 0.48^abc^	13.25 ± 0.32^a^	2.72 ± 0.01^a^	1.69 ± 0.05^a^	1.14 ± 0.03	11.78 ± 0.55^b^	2.64 ± 0.44
LCON	61.39 ± 0.12^a^	16.33 ± 0.49^abc^	13.34 ± 0.19^a^	2.70 ± 0.04^a^	1.56 ± 0.06^a^	1.16 ± 0.03	9.99 ± 0.22^b^	2.92 ± 0.39
*p*‐value	**<0.001**	**0.003**	**<0.001**	**<0.001**	**<0.001**	0.879	**<0.001**	0.067

*Note*: Values are means ± standard error of means. Means within the same column with the same letter are not significantly different (*p* < 0.05).

Abbreviations: 0.1B, 0.1% beet powder; 0.3B, 0.3% beet powder; 0.5B, 0.5% beet powder; CON, 156 ppm sodium nitrite; L0.0B, 6% lentil flour; L0.1B, lentil flour with 0.1% beet powder; L0.3B, lentil flour with 0.3% beet powder; L0.5B, lentil flour with 0.5% beet powder; LCON, lentil flour with 156 ppm sodium nitrite; ns, no significant difference among treatments (*p* > 0.05).

*P*‐Values in bold indicate statistically significant results.

### 3.2 Emulsion stability

**FIGURE 1:**
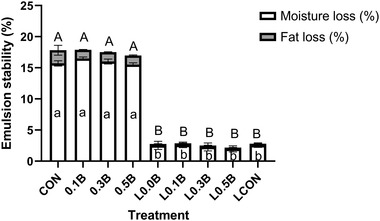
Emulsion stability of the pork batters formulated with nitrite, lentil flour, and three levels of beet powder. CON, 156 ppm sodium nitrite; 0.1B, 0.1% beet powder; 0.3B, 0.3% beet powder; 0.5B, 0.5% BP; L0.0B, 6% LF; L0.1B, LF with 0.1% BP; L0.3B, LF with 0.3% BP; L0.5B, LF with 0.5% BP; LCON, LF with 156 ppm sodium nitrite

Meat batters incorporated with LF showed lower total expressible fluid (*p* < 0.001), which is the sum of moisture and fat released from samples (Figure 1) after heating batters at 80°C for 1 h. Emulsion stability illustrates the ability of a meat emulsion to retain moisture and fat during processing. As was observed for expressible moisture of cooked bologna, the amount of moisture released from meat batters incorporated with LF during cooking was significantly lower than that of products without LF incorporation (1.77% and 15.49% moisture released, respectively). Fat loss of products without LF was significantly higher than that of products with LF. However, incorporation of BP did not have any impact on emulsion stability of pork bologna.

It seems that the viscosity of the raw batters showed negative relationship with fat loss, water loss, and total expressible fluid illustrating the importance of this parameter to emulsion stability. The amylose and amylopectin content of lentil starch may have contributed to the swelling power and gelatinization serving to immobilize the lipid droplets (Blazek & Copeland, 2008; Kaur et al., 2010). The consequence of high gel stabilization and water binding capacity resulted in the improvement of emulsion stability (Lee, 2015) due to the LF.

### 3.3 Color

#### 3.3.1 Thermal processing effect

**FIGURE 2:**
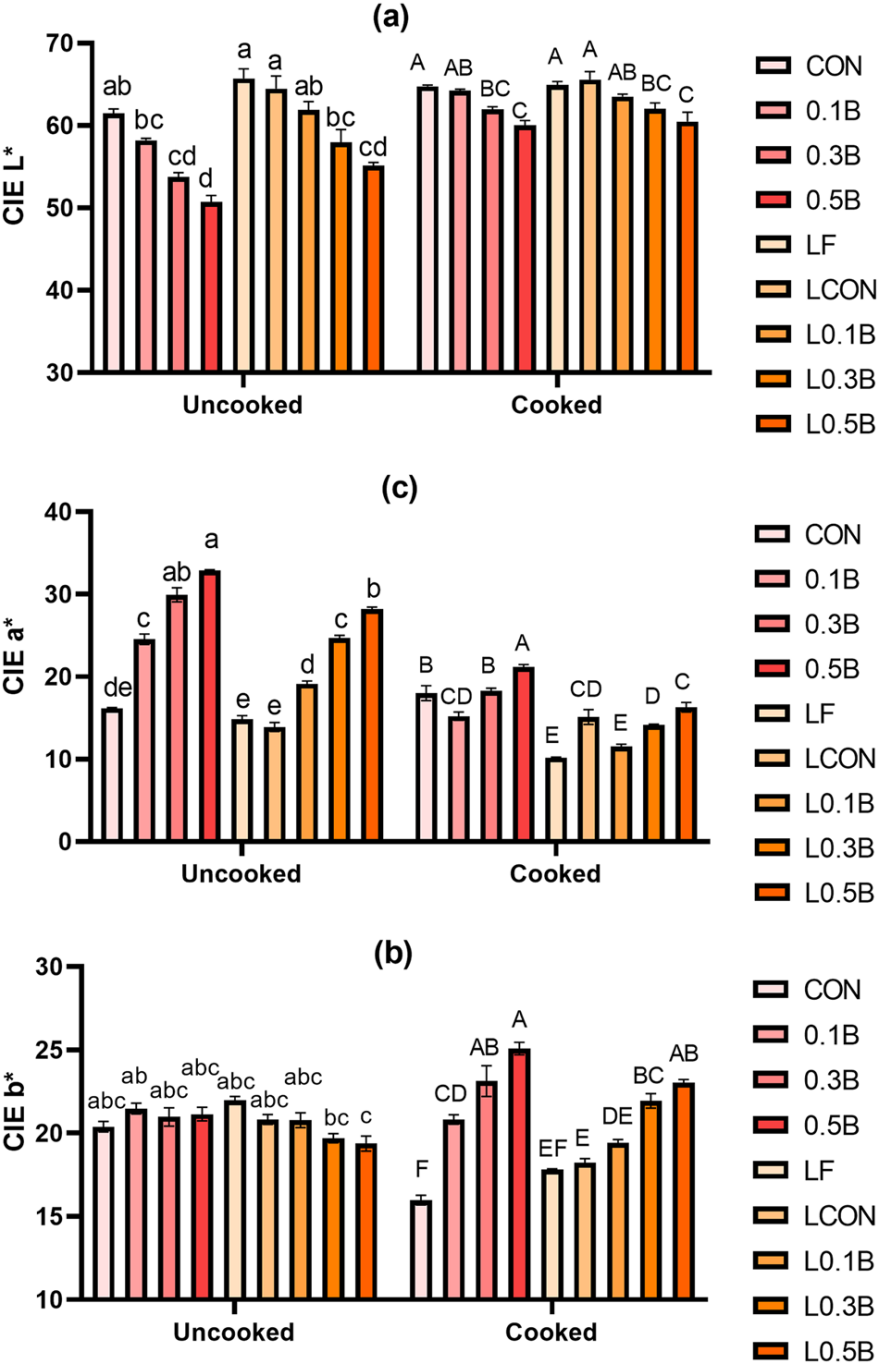
Color of raw and cooked pork bologna formulated with lentil flour, nitrite, and three levels of beet powder on day 0. (a) CIE L*, (b) CIE a*, and (c) CIE b*. Vertical bars show standard error of means. CON, 156 ppm sodium nitrite; 0.1B, 0.1% beet powder; 0.3B, 0.3% beet powder; 0.5B, 0.5% BP; L0.0B, 6% LF; L0.1B, LF with 0.1% BP; L0.3B, LF with 0.3% BP; L0.5B, LF with 0.5% BP; LCON, LF with 156 ppm sodium nitrite. Different small letters on the bars indicate significant difference between uncooked samples. Different capital letters on the bars indicate significant difference between cooked samples.

The color of the raw batters changed with cooking, but the extent of color changes differed by treatment, as evidenced by a significant interaction of treatment with cooking state for CIE L*, a*, and b* (Figure 2; *p* < 0.001). In the raw state, bologna with the addition of BP showed a significant decrease in lightness and increase in redness in a dose‐dependent matter, whereas BP did not affect yellowness of raw bologna samples. The LF addition increased the lightness and decreased the redness of the bologna alone and in the presence of BP. The pale natural color of LF contributed to the color differences in agreement with Baugreet et al. (2016).

In the cooked state, the redness of bologna with 0.3% BP was similar to the control (156 ppm nitrite added) bologna in the absence of LF (156 ppm sodium nitrite [CON] at 18.02 and 0.3% BP at 18.29). In the presence of LF, the redness of the bologna with 0.3% and 0.5% BP (14.15 and 16.30) was similar to the LF with 156 ppm sodium nitrite (LCON) bologna incorporated with 156 ppm nitrite (15.12). Thus, it was possible to match instrumental redness of cooked products with careful selection of BF addition level.

A desirable pink color remained in the nitrite‐added treatments after cooking since the nitrosohemochrome, which shows pink color in meat products, was formed due to the thermal processing (Honikel, 2008). Lightness of BP‐containing samples increased after cooking, with those with 0.1% and 0.3% BP now being similar to corresponding control and lentil control samples. Although the increase in the level of BP linearly increased the redness of raw samples, the cooking procedure highly reduced the redness of BP treatments. By contrast, the results showed that yellowness of BP treatments was enhanced after cooking. A similar result has been reported recently by Sakai et al. (2022) who have shown the decolorization problem of beet red when it is applied to plant‐based meat products. It is likely that the red pigments (betanin and isobetanin) of BP were degraded into yellow pigments (neobetanin and vulgaxanthin) due to the thermal processing (Herbach et al., 2004).

#### 3.3.2 Color stability during storage

**FIGURE 3:**
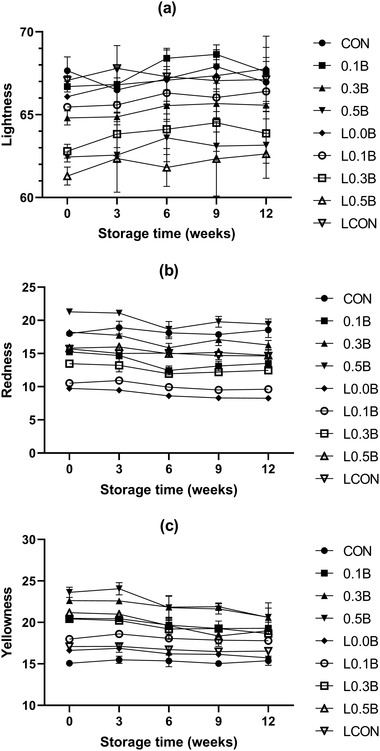
Changes in color (lightness, redness, and yellowness) of pork bologna formulated with lentil flour, nitrite, and three levels of beet powder during 4°C refrigerated storage for up to 12 weeks. CON, 156 ppm sodium nitrite; 0.1B, 0.1% beet powder; 0.3B, 0.3% beet powder; 0.5B, 0.5% BP; L0.0B, 6% LF; L0.1B, LF with 0.1% BP; L0.3B, LF with 0.3% BP; L0.5B, LF with 0.5% BP; LCON, LF with 156 ppm sodium nitrite. (a) CIE L*. (b) CIE a*. and (c) CIE b*

The color changes of vacuum‐packaged samples during refrigerated storage are shown in Figure 3. For lightness, there were significant treatment effects and storage time, but there was no interaction. The treatment effects on lightness have been described above. In addition, samples became lighter over storage time at 9 weeks.

There were significant treatment and storage time interactions in redness and yellowness (*p* < 0.05). Redness and yellowness values of nitrite‐added treatments were not significantly reduced over storage, whereas the values for BP treatments significantly decreased during storage in accordance with Jeong et al. (2010) who reported that emulsified sausage with added BF showed lower color stability than sausages with added nitrite. The redness was reduced by approximately 2.0 units in the absence of LF, whereas the redness was reduced by approximately 1.0 unit over storage time at 12 weeks when LF was added. Thus, the addition of LF helped to inhibit the reduction of redness during storage. In contrast, BF showed good color stability under the lighting display when it was used in raw noncured fresh sausages (Martínez et al., 2006). In cooked sausage, it is difficult to predict the final color expected as the thermal processing changes the color and may also diminish the color stability of BP. Our results showed that the nitrosohemochrome had a higher color stability than red pigments of BP in vacuum‐packaged meat products stored in the dark.

### 3.4 Torsion and TPA texture characteristics

**FIGURE 4:**
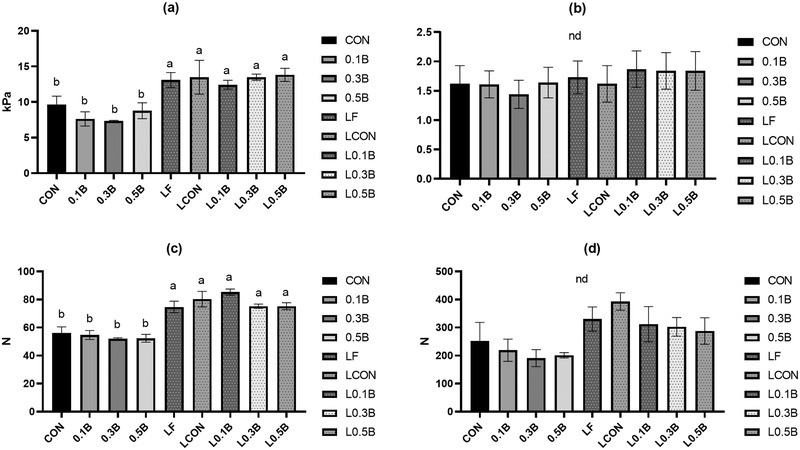
Torsional geometry (shear stress and strain at failure) and TPA texture characteristics of pork bologna formulated with lentil flour and three levels of beet powder: (a) Shear stress, (b) shear strain, (c) hardness, and (d) chewiness. Vertical bars show standard error of means. CON, 156 ppm sodium nitrite; 0.1B, 0.1% beet powder; 0.3B, 0.3% beet powder; 0.5B, 0.5% beet powder; L0.0B, 6% lentil flour; L0.1B, lentil flour with 0.1% beet powder; L0.3B, lentil flour with 0.3% beet powder; L0.5B, lentil flour with 0.5% beet powder; LCON, lentil flour with 156 ppm sodium nitrite. Figures with different letters on bars differ significantly (*p* < 0.05). nd, no significant difference

Torsional rheology measures two different parameters: gel strength or shear stress to failure and the elasticity or shear strain. The LF‐incorporated bologna sausages had a higher shear stress than non‐LF treatments (*p* = 0.001; Figure 4). These results are in agreement with the reported values of similar types of chicken bologna sausages incorporated with lentil and mechanically separated chicken (Pathiraja & Shand, 2018).

TPA hardness of samples incorporated with LF was significantly higher than that of other treatments (*p* < 0.001) in agreement with Motamedi et al. (2015) who reported that hardness of hamburgers incorporated with 8% and 12% LF increased. Therefore, the high protein content and gelatinized starch in LF might contribute to the increase in hardness of samples. No significant differences in springiness, cohesiveness, and chewiness were found among the samples (data not shown). The incorporation of BP at the levels used did not result in significant differences for texture properties (*p* > 0.05) in accordance with previous studies showing that BP addition to meat products did not influence texture properties (Jin et al., 2014; Sucu & Turp, 2018).

### 3.5 Storage effect on pH

There were no significant differences (*p* > 0.05) in pH due to treatment (*p* > 0.05), but pH significantly decreased (*p* < 0.001) during the 12 weeks of storage (Table 3 and Figure S1). Although organic acid accumulation was not measured, the reduction of pH might be caused by the action of indigenous lactic acid bacteria under anerobic conditions (de Almeida et al., 2015).

**TABLE 3:** Effect of lentil flour and beet powder on pH, TBARS (malondialdehyde mg/ kg of product), and carbonyl and sulfhydryl contents (nmol/mg protein) of pork bologna sausages formulated with mechanical separated pork during 12 weeks of storage time

	pH	TBARS	Carbonyl	Sulfhydryl
Treatment				
CON	6.46^nd^	0.23^bc^	0.45^c^	63.22^ab^
0.1B	6.23	0.37^a^	0.88^a^	51.01^c^
0.3B	6.34	0.34^ab^	0.69^b^	55.45^bc^
0.5B	6.31	0.28^abc^	0.69^b^	55.37^bc^
L0.0B	6.28	0.30^abc^	0.60^bc^	65.80^ab^
L0.1B	6.39	0.31^abc^	0.54^bc^	64.46^ab^
L0.3B	6.40	0.34^abc^	0.48^c^	71.30^a^
L0.5B	6.34	0.28^abc^	0.60^bc^	71.16^a^
LCON	6.46	0.21^c^	0.48^c^	72.51^a^
*p*‐value	0.054	**0.005**	**<0.001**	**<0.001**
*SEM*	0.02	0.02	0.04	2.11
Storage (weeks)				
0	6.46^a^	0.19^b^	0.48^c^	73.81^a^
3	6.47^a^	0.28^a^	0.42^c^	69.34^a^
6	6.35^b^	0.29^a^	0.49^c^	67.26^ab^
9	6.27^b^	0.31^a^	0.75^b^	60.33^b^
12	6.23^b^	0.36^a^	0.88^a^	47.75^c^
*p*‐value	**<0.001**	**<0.001**	**<0.001**	**<0.001**
*SEM*	0.04	0.02	0.06	3.24
Interaction				
*p*‐value	0.989	0.966	0.131	1.000

*Note*: Means within the same column with the same letter are not significantly different (*p* < 0.05).

Abbreviations: 0.1B, 0.1% beet powder; 0.3B, 0.3% beet powder; 0.5B, 0.5% beet powder; CON, 156 ppm sodium nitrite; L0.0B, 6% lentil flour; L0.1B, lentil flour with 0.1% beet powder; L0.3B, lentil flour with 0.3% beet powder; L0.5B, lentil flour with 0.5% beet powder; LCON, lentil flour with 156 ppm sodium nitrite; ns, no significant difference among treatments (*p* > 0.05).

*P*‐Values in bold indicate statistically significant results.

### 3.6 Storage effect on lipid and protein oxidation

#### 3.6.1 Thiobarbituric acid reactive substance

The level of TBARS in the vacuum‐packaged pork bologna stored in the dark at 4°C was measured throughout the storage time as an index of lipid oxidation (Table 3). Main effects of treatment and storage time were significant, but their interaction was not, as shown in the table. Control samples had the lowest TBARS values, illustrating the strong antioxidant ability of sodium nitrite and sodium ascorbate. The 0.1% BP treatment had a higher TBARS value than the nitrite controls (CON and LCON). The addition of 0.1%–0.5% BP or 6% LF or their combination did not show an antioxidant effect on TBARS. The lack of effect of 6% LF is in contrast to other studies by our group showing improved lipid stability in both raw and cooked meats (Pathiraja & Shand, 2018). Although significant changes in TBARS were observed over the 12 weeks of storage time (*p* < 0.001), the initial TBARS value was 0.19 MDA mg/kg meat, which slightly increased to 0.36 after 12 weeks of storage time (Figure S1), indicating that lipid oxidation in this meat system was minimal, despite the addition of mechanically separated pork to all treatments. Similar results were reported by Powell et al. (2019) who found that TBARS at 0.13–0.14 MDA mg/kg of vacuum‐packaged pork bologna sausage did not increase during 98 days of refrigerated storage.

#### 3.6.2 Carbonyl and sulfhydryl contents

Table 2 shows the effects of BP and LF on protein oxidation (carbonyl and sulfhydryl contents) of pork bologna during the refrigerated storage. Significant effects of treatments and storage on carbonyl contents were found (*p* < 0.001), but an interaction effect between treatments and storage time was not found. The products incorporated with nitrite and sodium ascorbate showed the lowest values of carbonyl contents. There was a significant reduction in carbonyl content with 0.3%–0.5% BP compared to 0.1% BP, but values were still higher than the CON, except the combination of LF and 0.3% BP that reduced carbonyl contents to a similar extent as the CON and LCON. Overall, carbonyl content of the bologna remained stable for 6 weeks of storage (Figure S1), but then increased steadily over the remaining storage time.

Bologna with LF incorporation showed significantly higher sulfhydryl contents than non‐LF‐added products (Table 3). Significant effects of treatments and storage on sulfhydryl contents were found (*p* < 0.001), but there were no interaction effects between treatments and storage time. Initial high sulfhydryl content in the products incorporated with LF could be due to the high free sulfhydryl content (31.0 µmol/g) in lentil protein (Ladjal‐Ettoumi et al., 2016). The sulfhydryl contents of all treatments significantly decreased with increasing storage time due to sulfhydryl oxidation and cross‐linking.

BP did not show a significant effect on sulfhydryl oxidation in the absence of LF. The LCON, LF with 0.3% BP, and LF with 0.5% BP maintained the highest sulfhydryl contents, indicating less protein oxidation occurred, which is the first such report related to LF efficacy in delaying protein oxidation in cooked meat products. BP showed poorer antioxidant ability than other natural antioxidants against protein oxidation in meat systems (Račkauskienė et al., 2015).

The increase in carbonyl and decrease in sulfhydryl contents are due to the oxidation of proteins in meat products during storage. Primary and secondary oxidation products act as substrates for protein oxidation, with MDA formation being related to carbonyl formation and sulfhydryl treatment loss (Leygonie et al., 2012; Soyer et al., 2010). A protein radical, which is generated from oxidative stress, can affect the formation of protein hydroperoxide and carbonyl compounds through some amino acid‐side chain modification, while the protein radical directly accelerates the formation of disulfide bonds, resulting in the reduction of the sulfhydryl contents (Lund et al., 2011). In comparison with nitrite addition, overall, BP and LF addition showed weak antioxidant effects against protein oxidation.

### 3.7 Consumer sensory evaluation

Selected demographic information such as gender, age, education, consumption pattern, and perception for nitrite were queried in the survey (Tables S1 and S2). For the first part of the evaluation, samples were pre‐sliced and vacuum packaged to simulate commercial meat product display to identify visual perception of consumers of bologna when viewing a packaged product. Consumer color acceptability and their color descriptions are presented in Table 4. In the presence of nitrite, external and internal colors of bologna without LF were described as bright pink (4.70 and 4.80), whereas the samples with added lentil showed pale pink (3.80 and 3.98, respectively) in Figures S2 and S3.

**TABLE 4:** Effects of lentil flour and beet powder on color acceptability^1^ of packaged pork bologna samples (*n* = 60 panelists)

Treatments	External color	External acceptability	Internal color	Internal acceptability	Purge color	Purge acceptability
CON	3.88 ± 0.07^b^	4.17 ± 0.17^a^	3.92 ± 0.07^b^	4.23 ± 0.18^ab^	3.49 ± 0.14^b^	4.38 ± 0.16^a^
0.1B	3.05 ± 0.16^c^	3.92 ± 0.14^ab^	3.03 ± 0.17^c^	3.97 ± 0.14^cb^	3.15 ± 0.14^cd^	4.25 ± 0.14^a^
0.3B	4.27 ± 0.18^ab^	4.20 ± 0.14^a^	4.02 ± 0.13^b^	4.38 ± 0.14^ab^	4.87 ± 0.13^a^	3.17 ± 0.15^bc^
0.5B	4.82 ± 0.15^a^	4.00 ± 0.15^ab^	4.62 ± 0.13^a^	4.75 ± 0.12^a^	5.40 ± 0.12^a^	2.82 ± 0.18^bc^
L0.0B	1.70 ± 0.07^d^	2.65 ± 0.17^c^	1.68 ± 0.06^d^	2.75 ± 0.17^e^	2.13 ± 0.13^e^	3.45 ± 0.19^b^
L0.1B	1.53 ± 0.07^d^	3.07 ± 0.19^c^	1.45 ± 0.06^d^	3.13 ± 0.18^de^	2.52 ± 0.15^de^	3.47 ± 0.15^b^
L0.3B	2.03 ± 0.17^d^	3.20 ± 0.17^c^	1.67 ± 0.13^d^	3.28 ± 0.16^cde^	3.70 ± 0.22^bc^	3.20 ± 0.16^bc^
L0.5B	2.85 ± 0.22^c^	3.33 ± 0.16^bc^	2.67 ± 0.15^c^	3.60 ± 0.15^cd^	5.05 ± 0.18^a^	2.65 ± 0.17^c^
LCON	3.80 ± 0.11^b^	4.45 ± 0.12^a^	3.98 ± 0.08^b^	4.55 ± 0.11^ab^	3.57 ± 0.13^bc^	4.52 ± 0.13^a^
*p*‐value	**<0.001**	**<0.001**	**<0.001**	**<0.001**	**<0.001**	**<0.001**

*Note*: Means ± standard error of mean within the same column with the same letter are not significantly different (*p* < 0.05). Six‐point acceptability scale: 1 = *dislike very much*; 6 = *like very much*. Color descriptions for external color (surface color near the edge) and internal color: 6‐point scale where 1 = *gray brown*, 2 = *gray*, 3 = *dark pink*, 4 = *pale pink*, 5 = *bright pink*, and 6 = *bright red*.

Abbreviations: 0.1B, 0.1% beet powder; 0.3B, 0.3% beet powder; 0.5B, 0.5% beet powder; CON, 156 ppm sodium nitrite; L0.0B, 6% lentil flour; L0.1B, lentil flour with 0.1% beet powder; L0.3B, lentil flour with 0.3% beet powder; L0.5B, lentil flour with 0.5% beet powder; LCON, lentil flour with 156 ppm sodium nitrite.

*P*‐Values in bold indicate statistically significant results.

The internal color acceptability of bologna slices increased as the level of BP increased (*p* < 0.001). However, the LF addition reduced external and internal color acceptability. It was observed that red pigment was released in the purge and visible in packages with BP, whereas the addition of LF reduced the redness of the purge. Contrary to the results of external and internal color acceptability, the purge color acceptability decreased as the level of BF increased (*p* < 0.001). For this study, due to limited sample availability, only 60 panelists were recruited, which is in the lower range for panel size, but results show clear differentiation of samples by the panelists. Stone and Sidel (2004) described that at least 40 panelists are recommended for consumer preference testing in the laboratory environment. Ares et al. (2014) confirmed that 60–80 consumers gave results consistent with that from larger panel numbers. It should also be noted that the use of 6‐point hedonic scales (excluding a neutral category) was used for consumer preference testing to mirror the structure of the intensity scales used. In effect, the panelists were required to indicate their liking or dislike of the samples and were not allowed to opt out with a neutral option. An examination of the results indicated that acceptability scores were consistently below or above 3.5 suggesting that omission of the neutral category was not a major concern in this study. It is more common, however, to use odd‐numbered hedonic scales.

Consumers had a very negative perception of the red purge. The negative effect of BP on purge in packages of meat products with added BP was not reported previously. It is noted that prior sensory evaluation studies of BP addition to meat products were conducted with unpackaged samples to investigate general quality parameters (Jeong et al., 2010; Jin et al., 2014), whereas we included an evaluation of packaged products. Further, this study illustrates the strong dislike of such color in the purge. Ways to retain the colorant and purge in the sausage should be explored.

Table 5 shows the effects of LF and BP on general sensory attributes of cubed samples of bologna. The products with added 0.5% BP (0.5% BP and L0.5B) were not tasted to limit the number of samples tasted (7) and due to the strongest negative perception of purge color as the result of visual sensory test.

**TABLE 5:** Effects of lentil flour and beet powder on sensory attributes of pork bologna samples (*n* = 60 panelists)

Treatments	Color acceptability	Texture acceptability	Juiciness acceptability	Flavor acceptability	Overall acceptability	Purchase intent
CON	4.64 ± 0.13^ab^	4.03 ± 0.17^ab^	4.53 ± 0.13^a^	4.38 ± 0.16^ns^	4.20 ± 0.13^a^	3.49 ± 0.19^ab^
0.1B	4.23^bc^ ± 0.12	3.80 ± 0.15^b^	4.37 ± 0.13^ab^	4.33 ± 0.15	3.98 ± 0.14^ab^	3.27 ± 0.19^ab^
0.3B	4.67 ± 0.13^ab^	4.12 ± 0.16^ab^	4.45 ± 0.14^ab^	4.32 ± 0.15	4.02 ± 0.16^ab^	3.35 ± 0.19^ab^
L0.0B	2.85 ± 0.15^e^	4.37 ± 0.14^ab^	4.25 ± 0.15^ab^	4.37 ± 0.17	3.92 ± 0.16^ab^	3.35 ± 0.19^ab^
L0.1B	3.12 ± 0.15^de^	4.20 ± 0.15^ab^	4.23 ± 0.14^ab^	4.13 ± 0.19	3.73 ± 0.16^b^	3.40 ± 0.19^ab^
L0.3B	3.70^c^ ± 0.15^d^	4.00 ± 0.17^ab^	3.95 ± 0.15^b^	4.05 ± 0.21	3.95 ± 0.17^ab^	3.12 ± 0.21^b^
LCON	4.78 ± 0.12^a^	4.63 ± 0.12^a^	4.45 ± 0.12^ab^	4.50 ± 0.14	4.47 ± 0.12^a^	4.08 ± 0.18^a^
*p*‐value	**<0.001**	**0.008**	**0.017**	0.525	**0.005**	**0.012**

*Note*: Means ± standard error within the same column with the same letter are not significantly different (*p* < 0.05). Six‐point acceptability scales 1 = *dislike very much*, 6 = *like very much*. Five‐point scale for purchase intent: 1 = *definitely would not*, 2 = *probably would not*, 3 = *might or might not*, 4 = *probably would*, and 5 = *definitely would*.

Abbreviations: 0.1B, 0.1% beet powder; 0.3B, 0.3% beet powder; 0.5B, 0.5% beet powder; CON, 156 ppm sodium nitrite; L0.0B, 6% lentil flour; L0.1B, lentil flour with 0.1% beet powder; L0.3B, lentil flour with 0.3% beet powder; L0.5B, lentil flour with 0.5% beet powder; LCON, lentil flour with 156 ppm sodium nitrite; ns, no significant difference among treatments (*p* > 0.05).

*P*‐Values in bold indicate statistically significant results.

The color acceptability of bologna with 0.3% BP was similar to the CON. Although the addition of LF lowered color acceptability, the addition of BP or nitrite enhanced color acceptability in the meat system formulated with LF. Nevertheless, the color acceptability scores for bologna with 0.3% BP were still lower than that for samples with nitrite, despite showing similar instrumental redness values. Thus, it was concluded that the consumer visual perception for the products differed showing less acceptability than the nitrite control. The consumer visual perception might be affected by other color attributes derived from BP that increased yellowness and decreased lightness compared to the nitrite treatments.

Texture acceptability was not affected much by treatment. Texture acceptability of bologna with nitrite and lentil addition was significantly higher than that of the product with added 0.1% BP, while that for all other treatments were similar. Liking of the juiciness was generally not affected by treatment, except that the nitrite control showed a high juiciness acceptability compared to the lentil and 0.3% BF‐added treatment (*p* < 0.05). There was no negative effect on the flavor acceptability with the removal of nitrite and addition of BP or LP or their combination; however, sensory evaluation was only done on samples that were stored for about 2 weeks after production.

The nitrite control showed the highest overall acceptability compared to LF and 0.3% BP treatment (*p* < 0.01). Purchase intent of nitrite with lentil addition was significantly higher than that of LF and 0.3% BP addition. Color and texture seemed to have major impact on the overall preference and purchase intent. Elzerman et al. (2011) found that color has a greater influence on consumer preference compared to flavor and texture. The BP provided positive perception for color acceptability due to the increase in redness, while LF addition decreased color acceptability in the absence of nitrite. We concluded that the combination of BP and LF has the potential to replace nitrite to a certain extent, but not for all sensory characteristics derived from sodium nitrite in pork bologna sausage incorporated with MSP.

### 3.8 Principle component analysis

**FIGURE 5:**
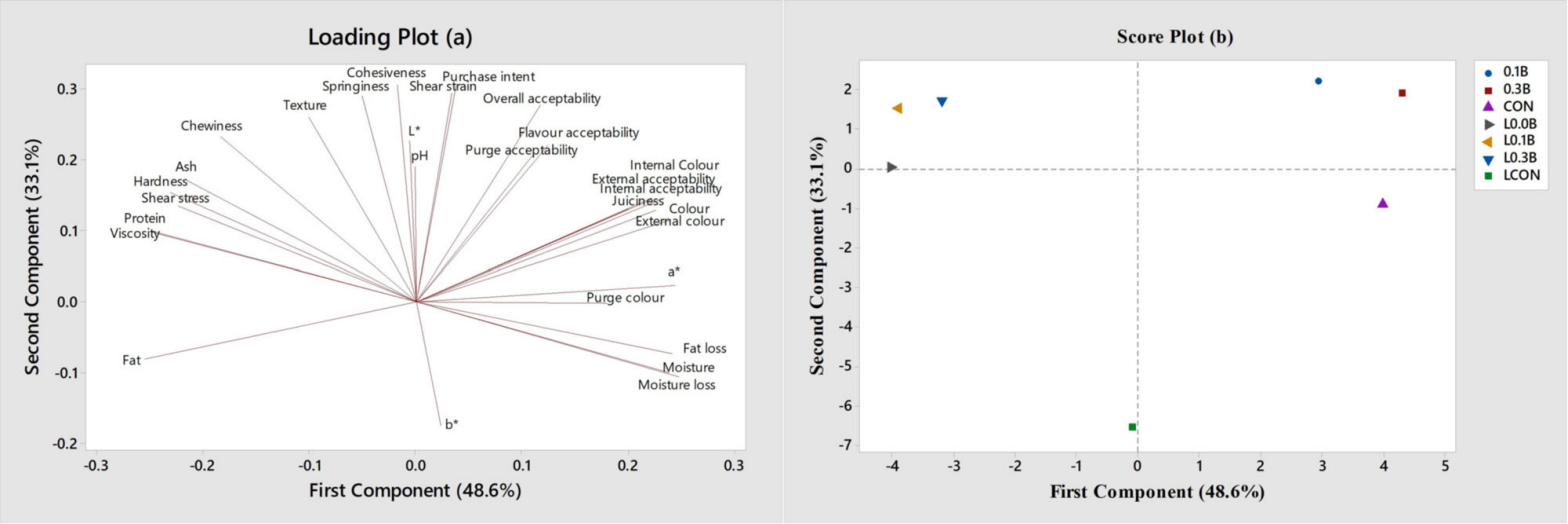
Loading plot (a) and score plot (b) of technological and sensory attributes of pork bologna formulated with lentil flour and beet powder in the PC1–PC2 space

Figure 5 show the results from a principal component analysis (PCA) of technological and sensory attributes. The first two principal components of loading plot accounted for 81.7% of the variability in the data (PC1, 48.6% and PC2, 33.1%). The loading plot illustrated several clusters: color‐related attributes (color intensities and acceptability) except purge acceptability were grouped. The purge acceptability was grouped with overall and flavor acceptability, showing its strong influence on overall liking. The purge color intensity was associated with redness (a*).

The score plot illustrates three clusters: first cluster formed with LF, LF with 0.1% BP, and LF with 0.3% BP, and second cluster formed with CON, 0.1% BP, and 0.3% BP. LCON was not clustered with other treatments. Therefore, it seemed that addition of 0.1%–0.3% BF to pork bologna can possibly replace color attributes derived from nitrite in the absence of lentil. However, the BF addition is not likely to replace the nitrite in pork bologna formulated with lentil, possibly due to the different paler pink color that resulted from the combination of BP with LF. Getting the right color when replacing nitrite is not easy, and it has been shown in this study that it is easily influenced by ingredient choices and levels, in addition to thermal processing (Domínguez et al., 2020; Güneşer, 2016).

## 4 CONCLUSION

This study revealed that the combination of BF and LF replaced some of the functionality of sodium nitrite in the pork bologna incorporated with MSP. The addition of 6% LF positively impacted oxidative stability, emulsion stability, and texture properties, while the addition of BF could express redness values close to products incorporated with sodium nitrite. The consumer sensory evaluations were conducted in two different approaches (1) to identify consumer visual perception when they shop for the packaged products and (2) to evaluate sensory characteristics. Particularly, we evaluated the consumer visual perception in the practical approach using vacuum‐packaged products to simulate a commercial product, which illustrated that consumers strongly dislike colored purge in the package that may occur with BP addition to meat products. Consequently, the usage of both components together had potential application as a sodium nitrite substitute, but a technical improvement will be required to reduce the red purge released from BP so as to increase consumer acceptability.

## AUTHOR CONTRIBUTIONS

Jihan Kim: Formal analysis; Investigation; Methodology; Software; Writing‐original draft. Phyllis J. Shand: Conceptualization; Funding acquisition; Project administration; Supervision; Validation; Writing‐review & editing.

## CONFLICT OF INTEREST

The authors declare no conflict of interest.

## Supporting information

Supplementary table 1. Demographics and consumer demand patterns for meat products of consumer sensory panelists (n = 60).
Supplementary table 2. Consumer panelist perceptions for sodium nitrite and willingness to pay (n = 60).
Supplementary figure 1. Changes in pH, TBARS, carbonyl and sulfhydryl concentrations of pork bologna formulated with lentil, nitrite and three levels of beet powder during 4°C refrigerated storage for up to 12 weeks.
Supplementary figure 2. Photographs of the pork bologna chops formulated nitrite, lentil flour and three levels of beet powder.
Supplementary figure 3. Photographs of the sliced and vacuum packaged pork bologna formulated nitrite, lentil flour and three levels of beet powder under display light.
