# Supplementary material for: Combined effect of beet powder and lentil flour as a partial nitrite substitute on physicochemical, texture and sensory characteristics, color, and oxidative stability of pork bologna
Source: J Food Sci. 2022 Sep 19;87(10):4379–93. doi: 10.1111/1750-3841.16302 (PMC9825927; doi:10.1111/1750-3841.16302)
Supplement: Supplementary file 1 — Supplementary table 1. Demographics and consumer demand patterns for meat products of consumer sensory panelists (n = 60). Supplementary table 2. Consumer panelist perceptions for sodium nitrite and willingness to pay (n = 60). Supplementary figure 1. Changes in pH, TBARS, carbonyl and sulfhydryl concentrations of pork bologna formulated with lentil, nitrite and three levels of beet powder during 4°C refrigerated storage for up to 12 weeks. Supplementary figure 2. Photographs of the pork bologna chops formulated nitrite, lentil flour and three levels of beet powder. Supplementary figure 3. Photographs of the sliced and vacuum packaged pork bologna formulated nitrite, lentil flour and three levels of beet powder under display light. [file JFDS-87-4379-s001.docx]

**Supplementary table 1.** Demographics and consumer demand patterns for meat products of consumer sensory panelists (n=60). Gender composition for this study was 42% male and 58% female. The majority of panelists were between the ages of 18-29 (73%) and 30-39 (20%) years. These panelists considered colour and price as the most important factors influencing meat buying decisions. Interestingly, absence of sodium nitrite was not regarded as a main selection factor compared to other factors. ^1)^ From 1, not at all to 6, extremely important ^a-c^ Means within the same column with the same letter are not significantly different (*P* < 0.05).

| **Response options** | **Frequency (%)** |
| --- | --- |
| **Gender** |  |
| Male | 41.67 |
| Female | 58.33 |
| **Age** |  |
| 18-29 | 73.33 |
| 30-39 | 20.00 |
| 40-49 | 3.33 |
| 50-59 | 1.67 |
| 60-69 | 1.67 |
| **Education** |  |
| High school (ordinary level) | 1.67 |
| High school graduate (advanced level) | 16.67 |
| University/college (Diploma) | 15.00 |
| University (Graduate) | 25.00 |
| Graduate school (Postgraduate) | 41.67 |
| **Consumer demand response patterns** | **Mean^1)^** |
| Price | 4.5^a^ |
| Absence of sodium nitrite | 3.1^b^ |
| Absence of mechanically separated meat | 2.76^c^ |
| Species (beef, pork and poultry) | 4.1^a^ |
| Colour | 4.6^a^ |
| Absence of preservatives | 3.78^ab^ |

^1)^ From 1, not at all to 6, extremely important ^a-c^ Means within the same column with the same letter are not significantly different (*P* < 0.05).

**Supplementary table 2.** Consumer panelist perceptions for sodium nitrite and willingness to pay (n=60). The processed meat consumption frequency was shown as 1-2 time per week, >3-5 times per week, and > 1-2 times per month. The majority of panelists preferred pale pink colour (45%) for sausages. Two thirds of the panelists knew the colourant effect of sodium nitrite in meat products. Additionally, the panelists commented about awareness of sodium nitrite in meat products as preservative, flavor and possible harmful effects. When asked if they were willing to buy meat products without added nitrite, the highest frequency (32%) indicated they would buy it even at the expense of lower colour acceptability.

| **Response options** | **Frequency (%)** |
| --- | --- |
| **How often do you consume processed meat products?** | |
| 3-5 times per week | 36.67 |
| 1-2 times per week | 40 |
| 1-2 times per month | 18.33 |
| Do not know meat consumption | 3.33 |
| **What color is preferred in sausages?** |  |
| Bright pink | 26.67 |
| Pale pink | 45.00 |
| Dark pink | 8.33 |
| Red | 8.33 |
| Grey Brown | 11.67 |
| **Are you aware that nitrites are added in some meat products to give their pink color?** | |
| Yes / No | 66.67 / 33.33 |
| **Are you aware that nitrite added in the products in listed under ingredients?** | |
| Yes /No | 65.00 / 35.00 |
| **If meat products without added nitrite are available in the market, what would be your purchasing choice?** | |
| I will buy it even at the expense of lower color acceptability. | 31.67 |
| I will buy it only if it still has pink color | 21.67 |
| I will buy it only if it is cheaper than the sausages with nitrites | 20.00 |
| I will buy any product without considering the presence or absence of added nitrites | 26.67 |


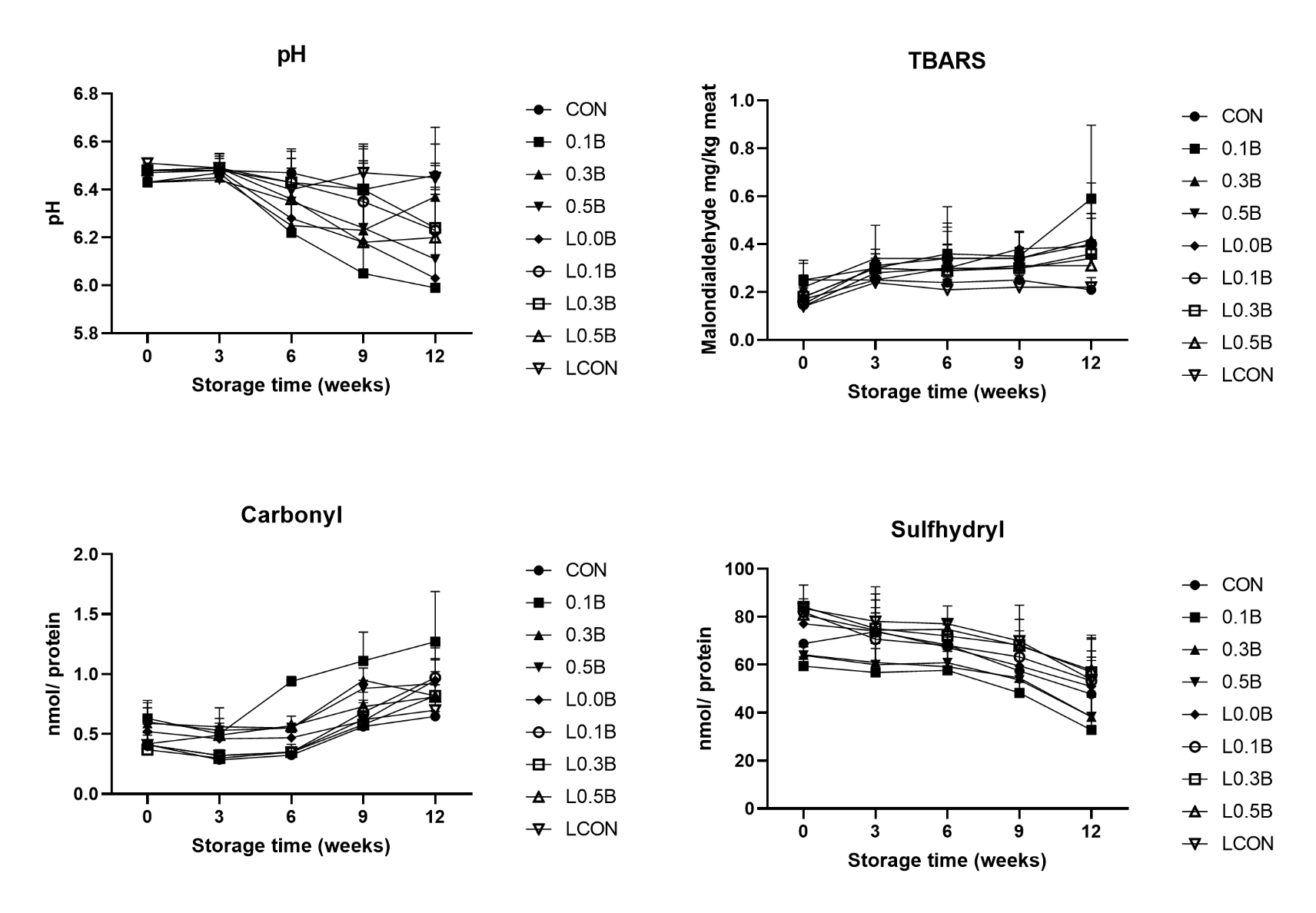


**Supplementary figure 1.** Changes in pH, TBARS, carbonyl and sulfhydryl concentrations of pork bologna formulated with lentil, nitrite and three levels of beet powder during 4°C refrigerated storage for up to 12 weeks. CON, 156 ppm sodium nitrite; 0.1B, 0.1% beet powder; 0.3B, 0.3% beet powder; 0.5B, 0.5% BP; L0.0B, 6% LF; L0.1B, LF with 0.1% BP; L0.3B, LF with 0.3% BP; L0.5B, LF with 0.5% BP and LCON, LF with 156 ppm sodium nitrite.


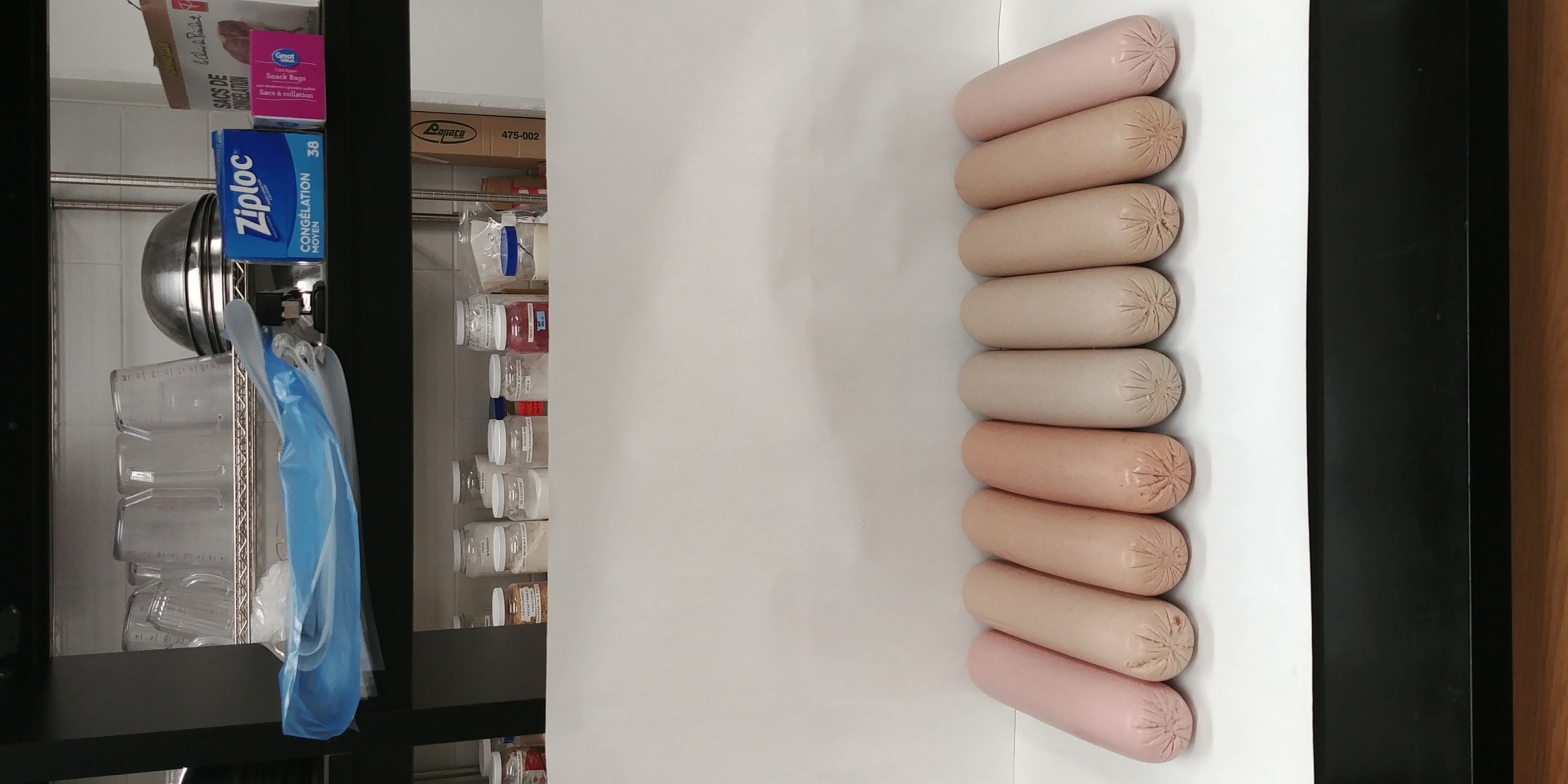


lentil flour

Non-lentil flour

156 ppm

nitrite

beet powder

0.1%

0.3%

0.5%

156 ppm

nitrite

beet powder

0.1%

0.3%

0.5%

0 %

**Supplementary figure 2.** Photographs of the pork bologna chops formulated nitrite, lentil flour and three levels of beet powder.


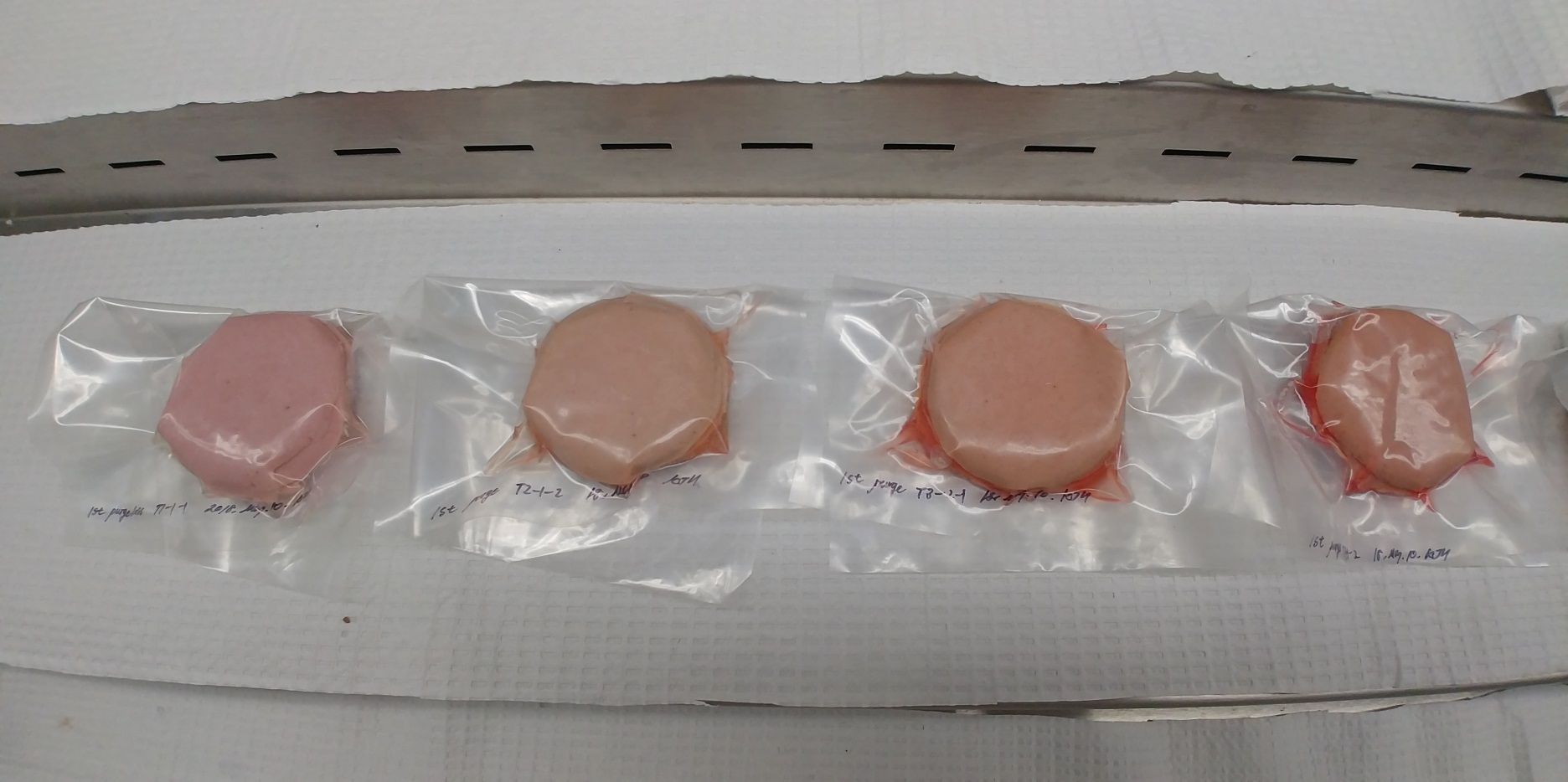

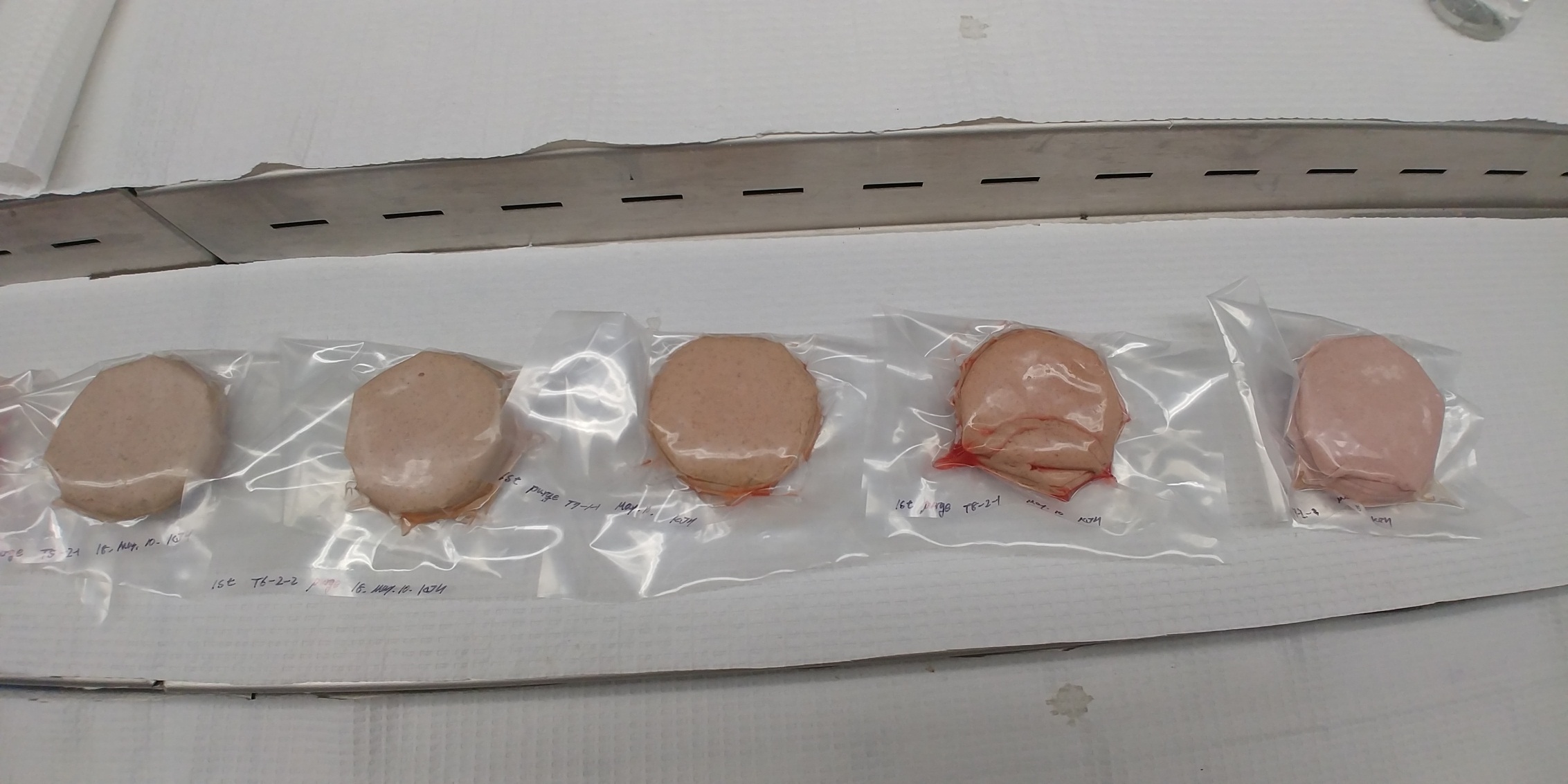


Non-lentil flour addition

6% Lentil flour addition

156 ppm nitrite

0.1% beet powder

0.3% beet powder

0.5% beet powder

0.1% beet powder

0.3% beet powder

0.5% beet powder

156 ppm nitrite

0% beet powder

**Supplementary figure 3.** Photographs of the sliced and vacuum packaged pork bologna formulated nitrite, lentil flour and three levels of beet powder under display light.
